# An explainable machine learning ensemble model to predict the risk of ovarian cancer in BRCA-mutated patients undergoing risk-reducing salpingo-oophorectomy

**DOI:** 10.3389/fonc.2023.1181792

**Published:** 2023-07-13

**Authors:** Maria Colomba Comes, Francesca Arezzo, Gennaro Cormio, Samantha Bove, Angela Calabrese, Annarita Fanizzi, Anila Kardhashi, Daniele La Forgia, Francesco Legge, Isabella Romagno, Vera Loizzi, Raffaella Massafra

**Affiliations:** ^1^ Struttura Semplice Dipartimentale di Fisica Sanitaria, I.R.C.C.S. Istituto Tumori “Giovanni Paolo II”, Bari, Italy; ^2^ Dipartimento di Medicina di Precisione e Rigenerativa e Area Jonica - (DiMePRe-J), Università di Bari “Aldo Moro”, Bari, Italy; ^3^ Ginecologia Oncologica, I.R.C.C.S. Istituto Tumori “Giovanni Paolo II”, Bari, Italy; ^4^ Dipartimento Interdisciplinare di Medicina (DIM), Università di Bari “Aldo Moro”, Bari, Italy; ^5^ Unità Operativa Semplice di Radiodiagnostica Avanzata, I.R.C.C.S. Istituto Tumori “Giovanni Paolo II”, Bari, Italy; ^6^ Struttura Semplice Dipartimentale di Radiologia Senologica, I.R.C.C.S. Istituto Tumori “Giovanni Paolo II”, Bari, Italy; ^7^ Unità di Ginecologia Oncologica, “F. Miulli” Ospedale Generale Regionale, Acquaviva delle Fonti, Bari, Italy; ^8^ Università di Bari “Aldo Moro”, Bari, Italy

**Keywords:** ovarian cancer, BRCA-mutation, risk-reducing salpingo-oophorectomy, machine learning, early identification

## Abstract

**Introduction:**

It has been estimated that 19,880 new cases of ovarian cancer had been diagnosed in 2022. Most epithelial ovarian cancer are sporadic, while in 15%–25% of cases, there is evidence of a familial or inherited component. Approximately 20%–25% of high-grade serous carcinoma cases are caused by germline mutations in the BRCA1 and BRCA2 genes. However, owing to a lack of effective early detection methods, women with BRCA mutations are recommended to undergo bilateral risk-reducing salpingo-oophorectomy (RRSO) after childbearing. Determining the right timing for this procedure is a difficult decision. It is crucial to find a clinical signature to identify high-risk BRCA-mutated patients and determine the appropriate timing for performing RRSO.

**Methods:**

In this work, clinical data referred to a cohort of 184 patients, of whom 7.6% were affected by adnexal tumors including invasive carcinomas and intraepithelial lesions after RSSO has been analyzed. Thus, we proposed an explainable machine learning (ML) ensemble approach using clinical data commonly collected in clinical practice to early identify BRCA-mutated patients at high risk of ovarian cancer and consequentially establish the correct timing for RRSO.

**Results:**

The ensemble model was able to handle imbalanced data achieving an accuracy value of 83.2%, a specificity value of 85.3%, a sensitivity value of 57.1%, a G-mean value of 69.8%, and an AUC value of 71.1%.

**Discussion:**

In agreement with the promising results achieved, the application of suitable ML techniques could play a key role in the definition of a BRCA-mutated patient-centric clinical signature for ovarian cancer risk and consequently personalize the management of these patients. As far as we know, this is the first work addressing this task from an ML perspective.

## Introduction

Ovarian cancer is a complex disease that led to 19,880 new cases and 12,810 deaths in 2022. According to the National Cancer Institute, it represents 1% of all new cancer cases and caused 2.1% of all cancer deaths (1). It is known as the “silent killer”, because it often has few early signs or symptoms and is not generally diagnosed until it has progressed to stage III or IV (2). According to the latest data from the National Institutes of Health (NIH), the 5-year survival rate for ovarian cancer is 49.7% (3). Most epithelial ovarian cancers are sporadic, while in 15%–25% of cases, there is evidence of a familial or inherited component. Approximately 20%–25% of all high-grade serous carcinoma cases are caused by germline mutations in genes called BReast CAncer gene 1 (BRCA1) and BReast CAncer gene 2 (BRCA2) (4). The BRCA1 and BRCA2 genes are human tumor suppressor genes that belong to the DNA damage repair pathway. They help to repair damaged DNA and play a role in ensuring the stability of the genome. BRCA1 and BRCA2 mutations are inherited in an autosomal dominant pattern (5). Therefore, offspring of an individual with one of these hereditary syndromes have a 50% chance of inheriting the pathogenic or likely pathogenic variant (6). Mutations in these genes have been linked to an increased risk of developing certain types of cancer (7). Women who carry a BRCA1 mutation have a 48.3% (95% CI, 38.8%–57.9%) cumulative risk of ovarian cancer by age 70, whereas carriers of a pathogenic BRCA2 variant have a 20.0% (95% CI, 13.3%–29.0%) cumulative risk by age 70 (8). The UK Collaborative Trial of Ovarian Cancer Screening highlights the importance of surveillance for BRCA1/2 mutation carriers based on transvaginal ultrasound and CA-125 starting at 30 years of age. However, after 11 years of follow-up, no significant decrease in mortality has been seen (9). Because of the lack of reliable early detection methods and the poor outcome associated with advanced ovarian cancer, in women who carry a BRCA mutation, bilateral risk-reducing salpingo-oophorectomy (RRSO), performed after childbearing, must be recommended (10). By removing these organs, the risk of developing these types of cancer can be reduced up to 90% (11). The fimbriae or distal tube is the most common site of origin for early malignancies found. Serous tubal intraepithelial carcinoma (STIC) is a lesion that affects only the epithelium of the fallopian tube (12, 13). It is considered to be an early precursor for serous ovarian cancer, and it is detected in 5%–8% of cases from patients carrying a pathogenic BRCA1/2 variant, who underwent RRSO (14). As STIC has been found in individuals who underwent surgery for risk reduction, the incidence and significance of these early lesions in the general population are uncertain (15). The correct age for performing RRSO remains a topic of debate. The National Comprehensive Cancer Network (NCCN) Guidelines Panel recommends RRSO between the ages of 35 and 40 in women with a BRCA1 variant, while women with BRCA2 mutation may delay the surgery until they are between 40 and 45 years old, unless earlier surgery is advised based on the age of diagnosis in the family (8). Furthermore, the manifestations of this hereditary syndrome can vary greatly among individuals within the same family, such as the age at which symptoms appear, the location of the tumor, and the number of primary tumors (16). This decision must consider multiple factors, including the impact on reproduction; breast and ovarian cancer risk; the risks of premature menopause such as osteoporosis, cardiovascular disease, cognitive changes, vasomotor symptoms, and sexual concerns; and other medical considerations, which can also affect the woman’s emotional health (17). Moreover, occasionally, ovarian cancer cases occur in younger age groups than those recommended for RRSO.

In recent years, artificial intelligence and its machine learning (ML) branch have been widely applied to develop decision support systems with the purpose of solving challenging diagnosis and prognosis tasks within the biomedical field (18, 19). Their spread runs parallel to the willingness of clinicians to understand more about these “black box” systems, whose underlying functioning is ruled by quite complex mathematical formulas (20). An intelligible explanation on how a specific decision is achieved by ML models plays a key role for an effective applicability in clinical practice. To address this growing need, the challenge to explain and clearly clarify the choice made by ML techniques has been just recently investigated through the introduction of the so-called eXplainable Artificial Intelligence (21, 22) (XAI).

Within this emerging scenario, despite the fact that a plethora of ML models have been designed either to support ovarian cancer diagnosis or to aid clinical decision-making processes in ovarian cancer treatment plannings (23–25), there is a lack of research works focusing on ML models to identify BRCA-mutated patients at high risk of ovarian cancer and consequently establish the correct timing for RRSO. In this study, we wanted to make a first effort to develop an explainable ML model addressing this task by leveraging the information power of some clinical features referred to a cohort of 184 BRCA-mutated patients undergoing RRSO, which have revealed the occurrence of adnexal tumors, including invasive carcinomas and intraepithelial lesions, for the 7.6% of the entire cohort.

## Methods and materials

### Problem formulation and data collection

The study was conducted according to the guidelines of the Declaration of Helsinki and approved by the Ethics Committee of the Azienda Ospedaliera Policlinico Consorziale-University of Bari, Italy (protocol code 6398). We integrated clinical features into an ML model to predict the occurrence of ovarian cancer on a cohort of 184 BRCA-mutated patients who underwent RSSO at the Department of Gynecologic Oncology–University of Bari, Italy, in the period between May 2017 and April 2022. After RSSO, adnexal tumor occurrence has been shown in 7.6% of these patients. In particular, a STIC was found in 3.3% of patients, while a diagnosis of invasive ovarian cancer (stage IA–IC) was made in 4.3% of woman. Hence, the resulting dataset we treated was imbalanced. We formulated a binary classification task to distinguish patients for which ovarian cancer have or have not occurred. In the following, the label *rare class* will indicate those patients for whom ovarian cancer occurred, while the label *abundant class* will point out control cases.

**Table 1** summarizes the data collected from the patients’ medical records. They comprised both categorical and continuous variables, such as age at time of RRSO (abbr. Age), body mass index (abbr. BMI), age of menarche, BRCA 1 status (abbr. BRCA 1, values: Yes/No), BRCA 2 status (abbr. BRCA 2, values: Yes/No), serum CA-125 levels (UI/ml) preoperative (abbr. CA-125), menopause at time of RRSO (abbr. MatoRRSO, values: Yes/No), number of pregnancy normal full term delivery (abbr. Pregnancy nftd), estroprogestin use (values: Yes/No), history of endometriosis (values: Yes/No), previous abdominal/pelvic surgery (abbr. PAPS, values: Yes/No), previous breast cancer (abbr. Previous BC, values: Yes/No), status of breast cancer first-degree relatives (abbr. BC FDR, values: Yes/No), number of breast cancer first-degree relatives (abbr. BC Nfdr), status of breast cancer second-degree relatives (abbr. BC SDR, values: Yes/No), number of breast cancer second-degree relatives (abbr. BC Nsdr), status of ovarian cancer first-degree relatives (abbr. OC FDR, values: Yes/No), number of ovarian cancer first-degree relatives (abbr. OC Nfdr), status of ovarian cancer second-degree relatives (abbr. OC SDR, values: Yes/No), and number of ovarian cancer second-degree relatives (abbr. OC Nsdr). Thus, a total of 20 clinical characteristics were gathered. The few missing data (see **Table 1**) were estimated through a proximity algorithm applied on the entire dataset (26). Where a clinical feature of a patient *np* had missing value, the algorithm assigns the value of the same feature related to the patient without any missing feature values and whose feature vector comprising all those features without missing values for the patient *np* had the minimum Euclidean distance from the corresponding feature vector of the patient *np*. Finally, before data analysis, continuous features were standardized by removing the mean and scaling to unit variance.

**Table 1:** Clinical characteristics.

Features	Distribution	Features	Distribution
**Overall**	184; 100%	**BRCA 2**	
**Age**		No (abs.; %)	104; 56.5%
Median; [*q*_1_,*q*_3_]	50 [45, 56]	Yes (abs.; %)	80; 43.5%
NA (abs.; %)	4; 2.2%	**MatoRRSO**	428; 88.0%
**BMI**		No (abs.; %)	62; 33.6%
Median; [*q*_1_,*q*_3_]	24.2 [22.0, 27.2]	Yes (abs.; %)	104; 56.5%
NA (abs.; %)	17; 9.2%	NA (abs.; %)	18; 9.8%
**Age of menarche**		**Estroprogestin use**	
Median; [*q*_1_,*q*_3_]	12 [12, 13]	No (abs.; %)	125; 67.9%
**BRCA 1**		Yes (abs.; %)	59; 32.1%
No (abs.; %)	80; 43.5%	**History of endometriosis**	
Yes (abs.; %)	104; 56.5%	No (abs.; %)	181; 98.4%
		Yes (abs.; %)	3; 1.6%
**CA-125**		**BC Nfdr**	
Median; [*q*_1_,*q*_3_]	10.5 [7.0, 15.6]	Median; [*q*_1_,*q*_3_]	0 [0, 1]
**Pregnancy nftd**		NA (abs.; %)	12; 6.5%
Median; [*q*_1_,*q*_3_]	2 [1, 2]	**BC Nsdr**	
NA (abs.; %)	14; 7.6%	Median; [*q*_1_,*q*_3_]	0 [0, 1]
**PAPS**		NA (abs.; %)	12; 6.5%
No (abs.; %)	113; 61.4%	**OC FDR**	
Yes (abs.; %)	71; 38.6%	No (abs.; %)	141; 76.6%
**Previous BC**		Yes (abs.; %)	31; 16.9%
No (abs.; %)	78; 57.6%	NA (abs.; %)	12; 6.5%
Yes (abs.; %)	106; 42.4%	**OC SDR**	
**BC FDR**		No (abs.; %)	137; 74.5%
No (abs.; %)	116; 63.1%	Yes (abs.; %)	35; 19.0%
Yes (abs.; %)	56; 30.4%	NA (abs.; %)	12; 6.5%
NA (abs.; %)	12; 6.5%	**OC Nfdr**	
**BC SDR**		Median; [*q*_1_,*q*_3_]	0 [0, 1]
No (abs.; %)	94; 51.1%	NA (abs.; %)	12; 6.5%
Yes (abs.; %)	78; 42.4%	**OC Nsdr**	
NA (abs.; %)	12; 6.5%	Median; [*q*_1_,*q*_3_]	0 [0, 1]
		NA (abs.; %)	12; 6.5%

For categorical variables, absolute and percentage counts are reported in parentheses. For continuous variables, the median value and first (q_1_) and third (q_3_) quartiles of the distribution are indicated in squared brackets. The number of missing values (NA) is also specified.

### Multi-ensemble resampled model

**Figure 1:**
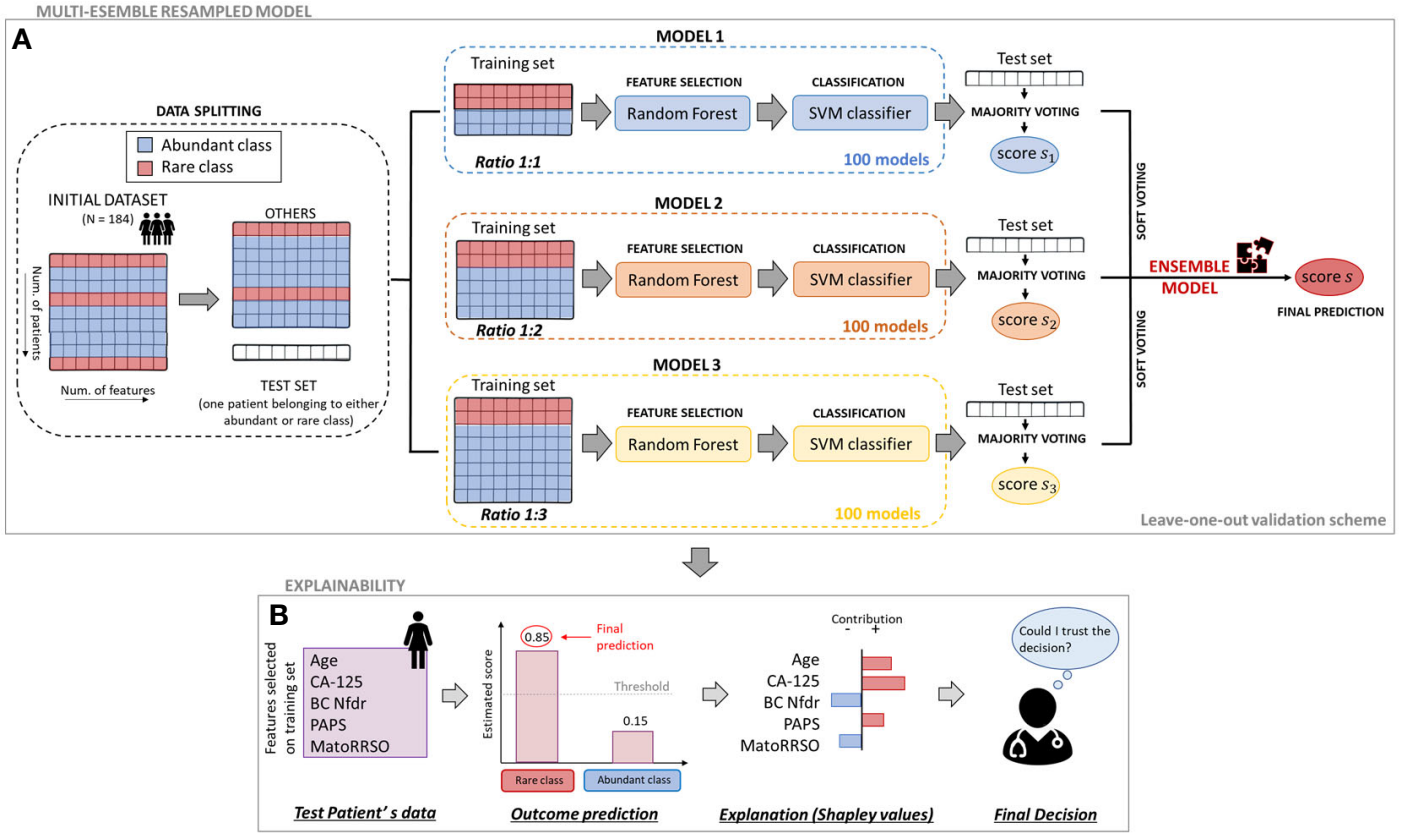
Workflow of the proposed approach. **(A)** Multi-ensemble resampled model. To estimate the classification score of each leaved-out patient, 100 models per ratio (ratio 1:1, ratio 1:2, and ratio 1:3) were defined. Their responses were firstly joined together by majority voting and then merged by soft voting. **(B)** Explainability. To evaluate the contribution of the analysed variables on the final prediction of a patient, an explainable model based on Shapley valuees computation was defined.

In this paper, we proposed an ML predictive model, which solved the above-mentioned classification task with the ability of handling imbalanced data. We called our proposal as *Multi-ensemble resampled model*, since it arose from the integration of two well-known techniques in the field, whose functioning is based on the under-sampling of the abundant class composing the initial dataset (27, 28). They are (i) *Ensemble Different Resampled Datasets* and (ii) *Resample with Different Ratios*. Technique (i) encompasses the building of *n* models, which employs all the *N* samples of the *rare class* and *N* randomly chosen samples of the abundant class. Then, all the *n* models’ predictions are jointed into an ensemble model to obtain a final single prediction per patient. Technique (ii) envisages the development of some models in which samples from the rare and abundant classes are resampled according to different ratios, e.g., ratio 1:1 (rare class:abundant class), ratio 1:2 (rare class:abundant class), and ratio 1:3 (rare class:abundant class). The ratio between the two classes could differently influence prediction (29). **Figure 1A** shows the workflow of the proposed model. As a first step, we split the initial dataset counting 184 patients into two datasets: one patient belonging to either abundant or rare class was left aside to be considered as test set; the set of “other patients” was resampled to build the training sets of some models falling in technique (ii). Each patient in turn was used as test set according to a leave-one-out validation scheme. In correspondence to each patient left out, 300 diverse models were designed. The training sets of these models were composed by a resampled set of “other patients” according to a diverse ratio between the classes, i.e., Model 1 with ratio 1:1, Model 2 with ratio 1:2, and Model 3 with ratio 1:3, respectively. Specifically, 100 models per ratio were developed. Two models related to a same ratio differed for the samples of the abundant class composing the training set, given that it was obtained by randomly choosing samples among all the samples of the abundant class of the “other patients” set (the random choice changed between the two models). When the test instance belonged to the abundant class, all the samples of the *rare class* fell into the training sets. Otherwise, the *rare class* of the training sets included all the samples of the same class except for the test instance. All the models we described before shared the same ML backbone structure. Once the training set was defined, a feature selection algorithm based on the Random Forest algorithm (30) was performed. According to a tree strategy with a configuration of 100 trees, the algorithm evaluates the so-called Gini impurity to identify the most important features. A weight of importance was then returned for each feature. One feature was deemed as important when its own weight was greater than the median weights computed over all the involved features. Afterwards, only the retained features on the training set were used to build a Support Vector Machine (SVM) classifier (31) with linear kernel, which gave the prediction on the test instance as output. The prediction was a classification score, whose values ranging between 0 and 1 are higher the greater the probability to belong to the *rare class*.

As a further step of analysis, the predictions returned for the test instance by all the 100 models per ratio were combined into a unique prediction per ratio (i.e., one prediction for ratio 1:1, another one for ratio 1:2, and one more for ratio 1:3) according to the rationale under technique (i). Specifically, a majority voting technique was performed (see **Figure 1A**). Each of the 100 models per ratio assigned the test instance to one class (abundant or rare class). The final class assignment per ratio corresponds to the class that was most frequently assigned by the 100 models. A unique classification score per ratio was also awarded to the test instance: it was computed as the maximum/minimum score of the models labeling the test instance into the rare/abundant class, if the class assigned by the majority voting was the rare/abundant class, respectively. At this point, three classification scores (one per ratio) were related to the test instance. To obtain a single classification score and, hence, a final class assignment, the three scores were averaged among them. In other words, a final Ensemble model based on a soft voting technique, which merged the predictions returned by ratio 1:1, ratio 1:2, and ratio 1:3 models after majority voting, was built. The code was implemented in ColabPro Notebook using Python programming language.

### Statistical analysis and performance evaluation

The association between each clinical feature and the classification label (abundant class *vs.* rare class) was evaluated by means of suitable statistical tests on all the datasets used to define the 100 resampled models per ratio. Specifically, the Wilcoxon–Mann–Whitney test (32) was performed for continuous features, whereas the chi-square test (33) was used for the clinical characteristics measured on an ordinal scale. A result was considered statistically significant when the *p*-value was less than 0.1. Standard metrics, such as area under the curve (AUC) as well as accuracy, sensitivity, specificity, and G-mean, were computed to evaluate model performances. While AUC measures the degree of separability of the classes predicted by the classifier, the other metrics, which were defined after the identification of the optimal threshold by Youden’s index on receiving operating characteristic (ROC) curve (34), are expressed with the following mathematical formulas:


Accuracy=(TP+TN)/(TP+TN+FP+FN)

Sensitivity=TP/(TP+FN)

Specificity=TN/(TN+FP)

$$\text{Gmean}=\sqrt{\text{Sensitivity}*\text{Specificity}}$$

where TP and TN stand for true positive (number of rare cases correctly classified) and true negative (number of abundant cases correctly classified), while FP (number of abundant cases misclassified as rare cases) and FN (number of rare cases misclassified as abundant cases) are false positive and false negative ones, respectively. The geometric mean (G-mean) is usually used to evaluate performances on imbalanced data (35) since, according to its definition, it balances between classification performances on both the classes. Both statistical analysis and performance evaluation were implemented using MATLAB R2022a (MathWorks, Inc., Natick, MA, USA) software.

### Explainability

As a final step of our analysis, we used a well-known XAI technique, based on SHapley Additive exPlanations (SHAP) values computation (36), to provide examples of *individual explanations*, i.e., explanations of the decision-making process underlying the implemented models to make a final decision for individual patients. An individual explanation could be finally evaluated by clinicians to make an informed decision on whether to trust the model’s prediction for that patient (**Figure 1B**). Basically, a SHAP value, which is a numeric value, is assigned to each feature related to a specific subject. According to the value that assumed for the specific patient, a feature either contributes to increment the risk of ovarian cancer (SHAP value with a positive sign) or does not (SHAP value with a negative sign). The SHAP absolute value of a feature is higher the greater its weight to the final prediction, i.e., score classification. Each feature contribution is finally obtained by measuring the feature weight according to the principles of game theory, namely, when it is considered alone as well as in cooperation with all the other features involved (36, 37). These SHAP values are estimated *via* a local-agnostic algorithm, which, regardless which concepts the classifier under study apprehends, it learns *via* an interpretable linear model at local decision level, i.e., for each test patient, by processing only the input and the output of a classifier. More specifically, the contribution of each feature value related to the *i*th test instance, is computed with respect to the *prediction difference*, that is, the difference between the score assigned by the classifier to the *i*th test instance and the so-called *base value*, which refers to the expected prediction if all the feature values are not known and is defined as the global average prediction over the training set. As a result, the algorithm returns one feature importance vector per test instance, which could vary from patient to patient, when that patient is considered as the *i*th test instance. For mathematical details, please refer to (36, 37).

## Results

### Feature importance

**Figure 2:**
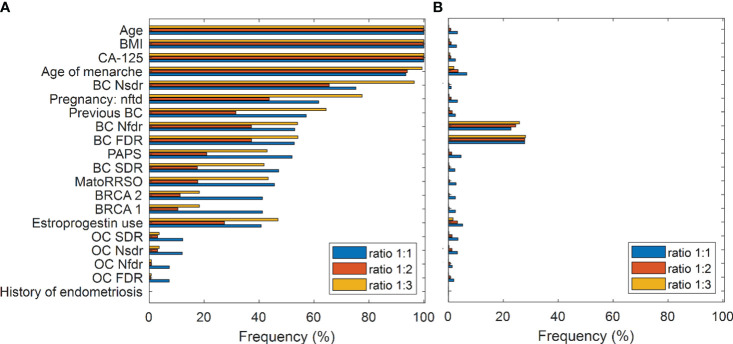
Feature importance. **(A)** Feature selection by means of Random Forest. Frequency of the features in all the diverse ratio models. Features are ranked according to their importance with respect to the Model 1 (ratio 1:1). **(B)** Statistical analysis result. Frequency of statistically significant features (*p*-value< 0.1) resulting from all the diverse ratio models. Mann–Whitney test and chi-square test are performed for continuous and categorical variables, respectively.

The assignment of a test instance to a class by means of the *Multi-ensemble resampled model* we proposed here relies on the concept of voting among 100 models for each of three ratios, ratio 1:1, ratio 1:2, and ratio 1:3. For each ratio, the statistical frequency of the features selected by means of the Random Forest algorithm over the training sets of the 100 models developed for each test instance was joined together to obtain an overall statistical frequency of the features for each ratio. **Figure 2A** highlights feature importance following feature selection by Random Forest for the three ratios. Features are ranked according to their importance with respect to Model 1 (ratio 1:1). Three features, namely, age at time of RRSO (abbr. Age), body mass index (abbr. BMI), and serum CA-125 levels (UI/ml) preoperative (abbr. CA-125), were selected with a frequency equal to 100% for each of the ratios. Two other features, Age of menarche and number of breast cancer second-degree relatives (abbr. BC Nsdr), had a frequency greater than 60% for each of the three ratios. The feature number of pregnancy normal full-term delivery (abbr. Pregnancy nftd) reached a frequency almost equal to 80% for ratio 1:3. While it exceeded the 60% frequency for ratio 1:1, it slightly reached a 40% frequency for ratio 1:2. The variable previous breast cancer (abbr. Previous BC), instead, exceeded the 60% frequency only for ratio 1:3. The other clinical characteristics related to breast cancer reached a frequency in the range [25%, 55%]. The features previous abdominal/pelvic surgery (abbr. PAPS), menopause at time of RRSO (abbr. MatoRRSO), and estroprogestin use were more important for ratio 1:3 and 1:1 rather than ratio 1:2. In addition, BRCA 1 and BRCA 2 reached a higher frequency with respect to ratio 1:1. The frequency in correspondence to all the features related to ovarian cancer was less than 20% for each of the ratios. Finally, as expected, the history of endometriosis feature has never been considered as important due to its little variability into the distribution (see **Table 1**). We compared feature importance obtained by applying the Random Forest algorithm, which evaluated feature importance according to a “multivariate” rationale, with those achieved by applying suitable “univariate” statistical tests on individual features depending on whether the variables are categorical or continuous (see Methods). In this case, feature importance was assessed according to the statistical significance of the features (*p*-value< 0.1) returned by the statistical tests. As emerged from **Figure 2B**, only two features, i.e., number of breast cancer first-degree relatives (abbr. BC Nfdr) and status of breast cancer first-degree relatives (abbr. BC FDR), achieved a frequency greater than 20% for each of the three ratios.

### Performance evaluation and explanation

**Figure 3:**
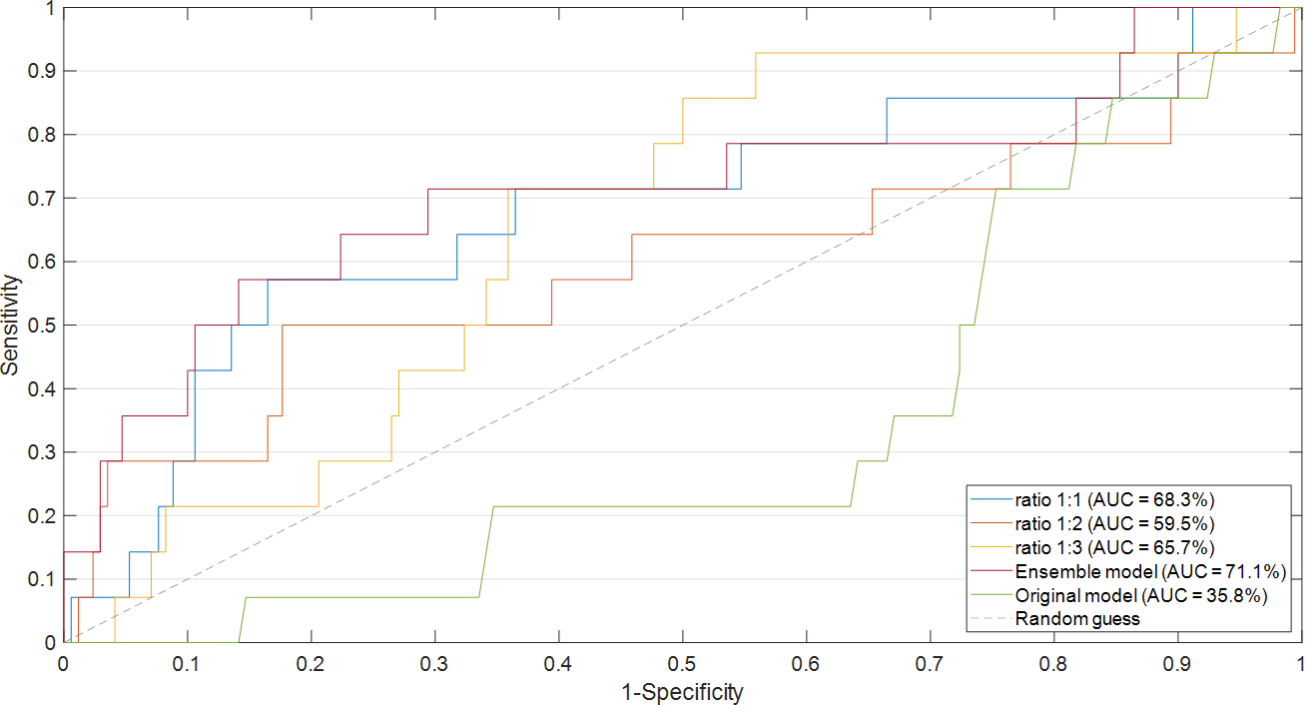
Comparison of ROC curve and the resulting AUC values. The ROC curves of the models obtained after applying majority voting for each of the three ratios were compared with the ROC curve of the Ensemble model. The ROC curve related to the original model was also drawn.

The proposed ensemble model achieved promising results: an accuracy value of 83.2%, a specificity value of 85.3%, a sensitivity value of 57.1%, a G-mean value of 69.8%, and an AUC value of 71.1%. **Figure 3** shows the ROC curve related to the Ensemble model (red curve) in comparison with the ROC curves related to the models obtained after applying majority voting for each of the three ratios. An AUC value of 68.3%, 59.5%, and 65.7% is reached in correspondence to ratio 1:1, ratio 1:2, and ratio 1:3, respectively. In addition, the ROC curve related to another model, indicated in the legend as the original model, has also been drawn in the figure. The original model represents a model, which has the same ML backbone structure of the models related to the diverse ratios (i.e., feature selection through the Random Forest algorithm and classification by means of SVM classifier), but made use of the entire dataset and a leave-out-one cross-validation scheme to evaluate predictions. The resulting AUC value was less than 0.5, which was much less informative with respect to the task we are solving. However, as well-known in the state of the art (38), it was expected due to the imbalance of our data. These results justify our choice to develop an *ad-hoc* system to handle imbalanced data.

**Figure 4:**
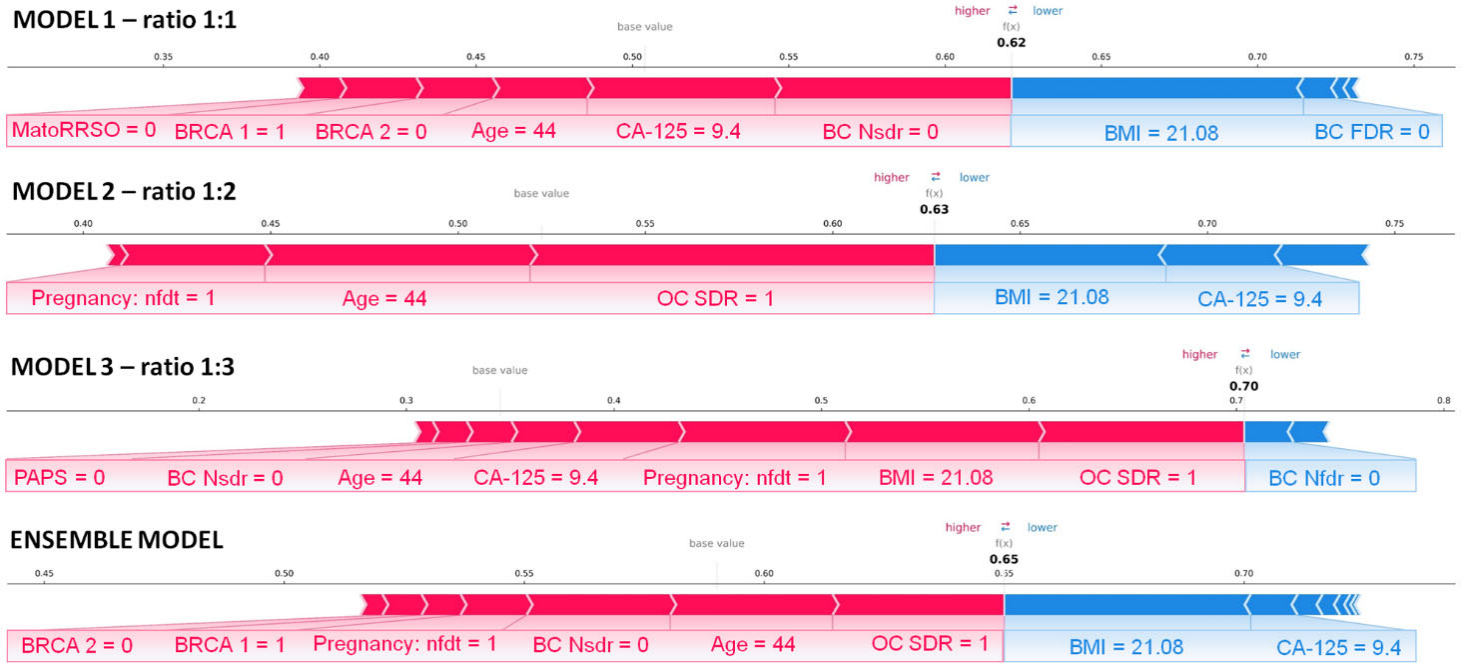
Individual explanation of a patient classified into the *rare class.* The explanations related to ratio 1:1, ratio 1:2, ratio 1:3, and the ensemble model are displayed. Representation of the additive SHAP values: the red color indicates a positive contribution, while the blue color indicates a negative contribution with respect to the score increase.

**Figure 5:**
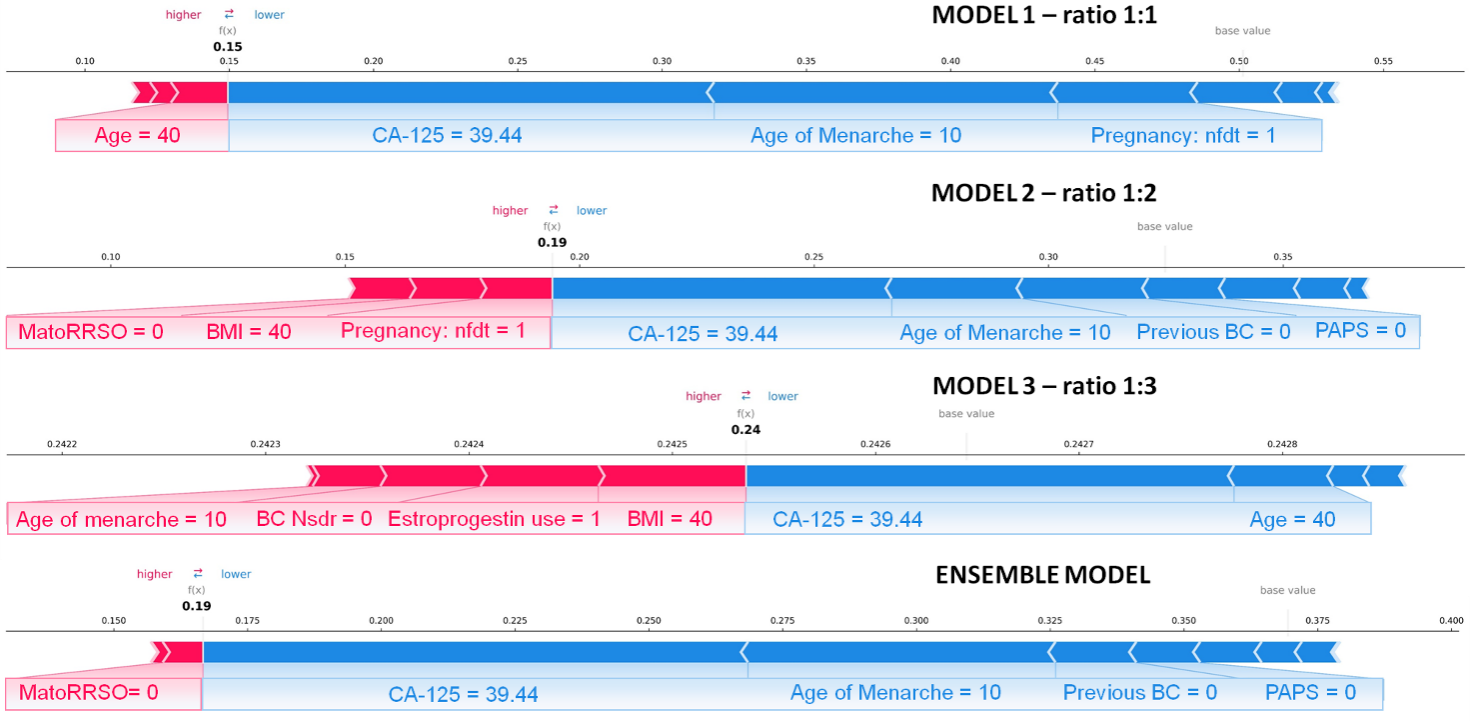
Individual explanation of a patient classified into the *abundant class.* The explanations related to ratio 1:1, ratio 1:2, ratio 1:3, and the ensemble model are displayed. Representation of the additive SHAP values: the red color indicates a positive contribution, while the blue color indicates a negative contribution with respect to the score increase.

Finally, we explored the application of XAI. **Figures 4**, **5** show individual explanations referring to a patient classified into the rare class and a patient classified into the abundant class, respectively. Only SHAP values of features with a greater weight as contribution to the final classification score are represented. Indeed, the number of both red and blue rows in the figures are in a greater number than the written features. Each figure highlights the individual explanations in correspondence to the three ratios, and even that obtained as a result of the ensemble model. For this last explanation, the classification score (0.65 in **Figure 4**; 0.19 in **Figure 5**) is the result of soft voting among the classification scores returned for the three ratios (0.62 for ratio 1:1, 0.63 for ratio 1:2, and 0.70 for ratio 1:3 in **Figure 4**; 0.15, 0.19, and 0.24 in **Figure 5**). Under the same rationale, the SHAP values related to the ensemble model were computed by averaging the SHAP values related to the three ratios. In this way, features with a SHAP value of opposite sign across the three models (e.g., Age in **Figure 5**, which has a positive sign for ratio 1:1 but a negative sign for ratio 1:3) had value near zero, when considered into the ensemble model (e.g., Age does not appear in the Ensemble model of **Figure 5**). It is worthy to remember that the SHAP values of each feature were computed by evaluating feature values in cooperation among them. For the patient portrayed in **Figure 4**, BRCA 2 = 0, BRCA 1 = 1, Pregnancy: nft = 1, BC Nsdr = 0, Age = 44, and status of ovarian cancer second-degree relatives (abbr. OC SDR) = 1 are the feature values, whose cooperation greatly contributes to increase the score, while BMI = 21.08 kg/m^2^ and CA-125 = 9.4 are the main features that decrease the risk. For the patient represented in **Figure 5**, on the one hand, the feature MatoRRSO tends to increase the score. The other red row stands for BMI. On the other hand, CA-125 = 39.44, together with Age of Menarche = 10, Previous BC =0, and PAPS = 0, decreases the score.

## Discussion

Determining the appropriate timing for performing RRSO is a challenging choice. Different series about pathologic findings at RRSO have shown that, in women with a pathogenic BRCA1/2 variant, 4.5% to 9% were found to have occult gynecologic neoplasia, including invasive carcinomas and intraepithelial lesions, through thorough pathologic examinations of their ovaries and fallopian tubes (39). This reveals that, despite RRSO being a crucial preventive-oncology strategy, it still has some failing. Therefore, identifying which women will derive the most benefit from RRSO and determining the optimal age at which surgery will provide maximum protection, while minimizing the negative effects of hormone deprivation, are essential (40). Patients with BRCA mutation are managed by thoroughly assessing their clinical characteristics, with the goal of detecting any signs of concern. In carriers of a pathogenic BRCA1 mutation, the prevalence of ovarian, fallopian tube, and peritoneal cancers found during RRSO was 1.5% for those under 40 years old and 3.8% for those between 40 and 49 years old. The highest incidence rate for carriers of a pathogenic BRCA1 mutation was between 50 and 59 years (annual risk of 1.7%) and that for carriers of a pathogenic BRCA2 mutation was between 60 and 69 years (annual risk of 0.6%). Thus, the recommended age for RRSO is earlier for women with a pathogenic or likely pathogenic BRCA1 mutation than for those with a BRCA2 mutation. Therefore, the largest number of studies recommend RRSO between the ages of 35 and 40 in women with a BRCA1 variant, while women with a BRCA2 mutation may delay the surgery until they are between 40 and 45 years old, unless earlier surgery is advised based on the age of diagnosis in the family (8). However, cases of cancer have been discovered in patients who are younger than the recommended age range. The ovarian cancer surveillance for BRCA1/2 mutation carriers recommend the evaluation of CA-125 level in combination with transvaginal ultrasound starting at 30 years of age (9). CA-125 is a protein that is often elevated in the blood of patients with ovarian cancer. High CA-125 levels have been linked to detection of cancer or dysplasia in RRSO (41). However, CA-125 is not specific to ovarian cancer, and elevated levels can be seen in other types of cancer and non-cancerous conditions as well (42). Obesity has been linked to an increased risk of certain types of cancer (43). Some studies have shown that higher BMI levels can be associated with an increased risk of developing ovarian cancer. Obesity can also impact the progression and outcomes of ovarian cancer, as well as the effectiveness of treatment options. Therefore, BMI is a clinical characteristic that should be considered in the classification and management of women with a BRCA mutation, but the evidence in this regard is not yet definitive (44). The need of finding a clinical signature to identify BRCA-mutated patients at high risk of ovarian cancer and consequentially establish the correct timing for performing RRSO is urgent. In clinical oncology, the relationship between patient characteristics and pathology findings is evaluated using both univariable statistical analysis and multivariable logistic regression. These models are based on the assumption of linear association. However, many clinicopathologic features could exhibit a more complex association in medicine. Thus, more sophisticated computerized algorithms, which are ruled by complex mathematical formulas, are developed within the ML branch to draw inferences from patterns in data (45–48).

To the best of our knowledge, this is the first work that tries to fulfill the task of giving an early diagnosis of ovarian cancer in BRCA-mutated patients before the execution of RSSO under an ML angle. Three peculiarities of the model could be underlined. On the one hand, the model functioning is ruled by multi-ensemble approaches, that, as already demonstrated in the state of the art (29), allow us to increase the robustness of the decision-making process with respect to single models. On the other hand, as proven by the achieved promising results (AUC = 71.1%), the resampling techniques we have adopted enable us to define a more representative model of the task under study, thus greatly overcoming performances of the original model, which has been implemented by involving the entire dataset without resampling (AUC = 35.8%). Not less relevant, the implementation of feature importance is the first attempt to globally explore the value of each feature with respect to the prediction, if considered in cooperation with the others on a set of patients (training sets). In addition, beyond a global evaluation of feature importance, we applied an XAI technique to investigate the local value of each feature, whose contribution was weighted, according to its importance, with all the other features on an individual patient. In the vein of personalized medicine, the evaluation of feature importance at the local level represents the first step towards the finding of a reliable patient-centric clinical signature.

This study represents a first effort to develop an explainable ML model addressing this task by leveraging the information power of some clinical features referred to a cohort of BRCA-mutated patients undergoing RRSO. However, one limitation of this work is the non-involvement of external validation data, i.e., data provided by other cancer centers. The feasibility as well as generalizability of the proposed model should be further validated on wider cohorts of patients. As the next step of our research work, we intend to apply the model on a cohort of patients, whose data will be yielded by multiple cancer centers and/or hospitals across Italy. This manuscript provides a first attempt to help clinicians categorize BRCA-mutated patients according to their risk of developing ovarian cancer. Although guidelines recommend prophylactic adnexectomy for BRCA1/2 mutation carriers within certain age ranges, there are instances where tumors arise at an earlier age or where patients never develop ovarian cancer at all. These variations can be attributed to diverse factors that influence cancer development. The foundation of the algorithm explored the intricate interplay between multiple patient variables, with a particular focus on understanding their non-linear associations (8). Despite the fact that results achieved are promising and intelligible, the model is not suitable to be applied in the actual clinical practice. In particular, the specificity and sensitivity values obtained are not balanced between them, as expected due to the use of an imbalanced dataset, which, anyway, respects the “real life” ratio between the two classes (abundant *vs.* rare). One future challenge will be to improve the mathematical rules underpinning the model to enhance sensitivity and, as a consequence, accuracy and AUC values. The integration of a wider cohort to analyze with higher performances, especially in terms of sensitivity, as well as with the explanation of the model’s decisions will be the key to develop a non-invasive tool, requiring few easy-to-collect attributes available at first visit, to be effectively used by clinicians. The power of such a tool will consist in identifying the features that could play a key role for the prediction of a specific patient and that can vary from patient to patient, thus defining a patient-centric signature within a global clinical signature. This is the major implication of the model findings on clinical practice recommendations among BRCA 1/2 mutation carriers and specifically on the individual person/patient based on varying personal or family history. However, at this step, in agreement with the goal of this work, we have started to observe the potentiality of the algorithm on two patients as examples (see **Figures 4**, **5**), by justifying the contribution of the features related to that patient to reach the obtained score representing the ovarian cancer risk.

In conclusion, the present study proposed an explainable ML model that exploits clinical data commonly collected in clinical practice to early identify which BRCA-mutated patients are more prone to an ovarian cancer risk and accordingly determine the appropriate timing for performing RRSO. The promising results achieved are valuable in suggesting how this work could represent the first building block towards the definition of an ML-based decision support system that could be effectively applied in clinical practice. Indeed, our ambitious purpose is to supply to clinicians a user-friendly tool that could return a score representing the ovarian cancer risk for each patient individually, with an intelligible explanation on how the clinical features taken into account for that patient could contribute to the estimated risk.

## Data availability statement

The raw data supporting the conclusions of this article will be made available by the authors, without undue reservation.

## Ethics statement

Ethical review and approval was not required for the study on human participants in accordance with the local legislation and institutional requirements. The patients/participants provided their written informed consent to participate in this study.

## Author contributions

Conceptualization, MC, FA, VL, GC, and RM. Methodology, MC and RM. Software, MC. Validation, MC, SB, AF, and RM. Formal analysis, MC, FA, VL, GC, and RM. Resources, RM. Data curation, IR, FA, DL, VL, and GC. Writing—original draft preparation, MC, FA, VL, GC, SB, AF, and RM. Writing—review and editing, MC, FA, VL, GC, SB, AF, DL, IR, FL, AK, AC, and RM. Supervision, GC, VL, and RM. All authors contributed to the article and approved the submitted version.
